# Objects and Action Detection of Human Faces through Thermal Images Using ANU-Net

**DOI:** 10.3390/s22218242

**Published:** 2022-10-27

**Authors:** Babu Rajendra Prasad Singothu, Bolem Sai Chandana

**Affiliations:** School of Computer Science and Engineering, VIT-AP University, Amaravathi 522237, India

**Keywords:** deep learning, thermal face recognition, thermal image, pre-processing, feature extraction, detection, face action classification

## Abstract

Thermal cameras, as opposed to RBG cameras, work effectively in extremely low illumination situations and can record data outside of the human visual spectrum. For surveillance and security applications, thermal images have several benefits. However, due to the little visual information in thermal images and intrinsic similarity of facial heat maps, completing face identification tasks in the thermal realm is particularly difficult. It can be difficult to attempt identification across modalities, such as when trying to identify a face in thermal images using the ground truth database for the matching visible light domain or vice versa. We proposed a method for detecting objects and actions on thermal human face images, based on the classification of five different features (hat, glasses, rotation, normal, and hat with glasses) in this paper. This model is presented in five steps. To improve the results of feature extraction during the pre-processing step, initially, we resize the images and then convert them to grayscale level using a median filter. In addition, features are extracted from pre-processed images using principle component analysis (PCA). Furthermore, the horse herd optimization algorithm (HOA) is employed for feature selection. Then, to detect the human face in thermal images, the LeNet-5 method is used. It is utilized to detect objects and actions in face areas. Finally, we classify the objects and actions on faces using the ANU-Net approach with the Monarch butterfly optimization (MBO) algorithm to achieve higher classification accuracy. According to experiments using the Terravic Facial Infrared Database, the proposed method outperforms “state-of-the-art” methods for face recognition in thermal images. Additionally, the results for several facial recognition tasks demonstrate good precision.

## 1. Introduction

**Figure 1:**
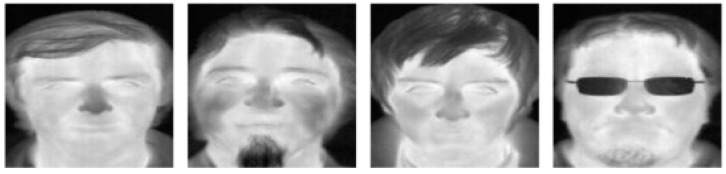
Example images of face recognition in thermal images.

Face recognition is one of the biometric qualities for security that has been most frequently used successfully in recent times. The study of biometrics and computer vision face recognition has proven to be one of the most difficult of topics. To solve the pose and illumination issues for visible face photos, many face recognition algorithms have been developed [1]. However, such a system’s thermal face images are susceptible to fluctuations in the surrounding temperature, which can result in identification errors. Infrared images work well in all weather and day/night situations to locate people from their surroundings depending on the radiation differential [2,3,4,5]. The primary concept behind thermal imaging is that each object produces infrared energy uniquely based on its properties and temperature. As a result, every object has a unique thermal signature. The pattern of the superficial blood vessels that are present beneath the skin of the face is the main source of this signature. Each person’s thermal image is distinct, since each face has a different vascular and tissue structure. There are many ways to find faces, from using image features to deep learning techniques. Facial recognition technology does not function properly without a reliable facial detection algorithm that is effective under different circumstances [6,7]. When face images are taken in a controlled setting, face identification based on the visible light spectrum has demonstrated good performance. However, under uncontrolled lighting circumstances, the effectiveness of such facial recognition systems is dramatically reduced. When the illumination is poor or the face is not evenly clear, the accuracy of face recognition rapidly decreases. Face identification is a difficult task in working with uncontrolled illumination when there are visible representations of the face [8,9,10,11]. In recent times, face recognition has utilized thermal images of the face. Figure 1 shows an example of face recognition thermal images.

Sometimes, face recognition in thermal images is a difficult process under various stances and lighting effects [12]. There are many methods to identify faces and classify them based on their expressions. However, these methods also have some difficulties in detecting faces. Detecting a face from thermal images has some issues and challenges. Higher dimensional datasets are required, to speed up the computation, and parallel algorithms can be employed. Illumination changes have little effect on face identification in thermal images. Moreover, when spectacles are visible in facial images, thermal imaging has very little success [13,14]. So, this paper classified the objects and actions in the face after detecting the face in thermal images. This is because detection of the face takes priority over finding the objects and actions on faces. This work proposed thermal images to enhance the performance of deep learning-based facial recognition systems. The goal of this work is to enable face recognition and classification to provide better outcomes. We proposed a method for detecting and classifying objects and actions, such as hats, glasses, normal, rotation, and hat with glasses, from human thermal face images based on a deep learning model in this work. This model has five steps; initially, images are resized, and the input thermal image is converted to grayscale image using the median filter in pre-processing. In addition, principle component analysis (PCA) is used to extract the features from pre-processed images. Furthermore, for feature selection, the horse herd optimization algorithm (HOA) is used. Then, the LeNet-5 technique is utilized to identify the human faces in thermal images for good classification results. Finally, we classify the objects and actions in face images, using the ANU-Net method combined with the Monarch butterfly optimization (MBO) algorithm to achieve a higher classification accuracy. The paper’s key contributions include:

➢ Resizing the images and grayscale thermal input images by using a median filter in the pre-processing stage.
➢ A principle component analysis (PCA) approach is proposed to extract the features of images. It extracts features such as glasses, a hat, or other specific objects from the pre-processed face image.
➢ The horse herd optimization algorithm (HOA) is used to select the features.
➢ A LeNet-5 technique is utilized to detect or identify the human face from thermal images. The detection of the face process is important to classify the objects and actions on the face.
➢ We used the ANU-Net methodology for the classification of objects and actions on faces in thermal images with the Monarch butterfly optimization algorithm to obtain higher accuracy.
➢ This paper proposed a method for detecting several face objects and actions in thermal images utilizing the Terravic Facial Infrared Database, a dataset of facial images for facial object recognition.

The remainder of the paper is ordered as follows. Section 2 presents the related works in the paper. The proposed methodology is shown in Section 3. In Section 4, the result is shown. Finally, in Section 5 the conclusion is presented.

## 2. Literature Survey

The most widely and effectively applied biometric technology in use today is face recognition. Holistic and texture-based methods are the two basic categories under which face recognition techniques can be divided. The complete facial image is taken as one signal and processed to obtain the necessary elements for classification using the holistic approach.

Kanmani and Narasimhan [15] suggested an Eigen face recognition system that benefits from the integration of visual and thermal facial pictures to increase the accuracy of face identification. The dual-tree discrete wavelet transform (DT-DWT) is the domain in which the first two fusion methods operate, whereas the CT is the domain in which the third method operates. To identify the best fusion coefficients, researchers use self-tuning particle swarm optimization (STPSO), particle swarm optimization (PSO), and brainstorm optimization algorithm (BSO) techniques.

Sancen et al. [16] presented a method for recognizing faces based on thermal pictures of inebriated faces. The Pontificia Universidad Católica de Valparaiso–Drunk Thermal Face database (PUCV-DTF) was utilized for the tests. The recognition system used local binary patterns (LBP) to operate. The bio-heat model’s depiction of thermal images and merging of those images with an extracted vascular network allowed for the extraction of the LBP features. The fused image and the LBP histogram of the thermogram with an anisotropic filter, respectively, are concatenated to create the feature vector for each image.

Nayak et al. [17] presented a three-stage HCI architecture to compute the multivariate time-series thermal video sequences and provide distraction. The first stage consists of following the face ROIs throughout the thermal video while simultaneously detecting faces, eyes, and noses using a Faster R-CNN architecture. The multivariate time-series (MTS) data is created by calculating the mean intensity of ROIs. The dynamic time warping (DTW) technique is used to categorize the emotional states produced by video stimulus in the second stage using the smoothed MTS data. In the third stage of HCI, our suggested framework offers pertinent recommendations from a physical and psychological distraction perspective.

Hu Li moments were suggested by Zaeri [18] as a technique for impaired thermal face identification. The approach addresses spatial variations in thermal imaging brought on by limited resolution and shifting head posture. They define a set of scalar values for thermal images that can capture their key characteristics at the component level. Each thermal image is broken down into its component statistical features, which are then fused using a fusion procedure. To provide an integrative result that is improved in terms of data content for accurate classification, the method combines some statistical patterns. They demonstrate that Hu Li moments used in local representations increase discriminability and give robustness against variability brought on by spatial changes.

Kezebou et al. [19] suggested a thermal to RGB generative adversarial network (TRGAN) to synthesize face images recorded in the thermal domain, to their RBG counterparts, to close present inter-domain gaps and considerably enhance cross-modal facial recognition skills. The suggested TR-GAN model almost doubles the cross-modal recognition accuracy when image translation is performed, according to experimental results on the TUFTS Face Dataset using the VGG-Face recognition model without retraining. It also outperforms other state-of-the-art GAN models on the same task.

## 3. Proposed Methodology

**Figure 2:**
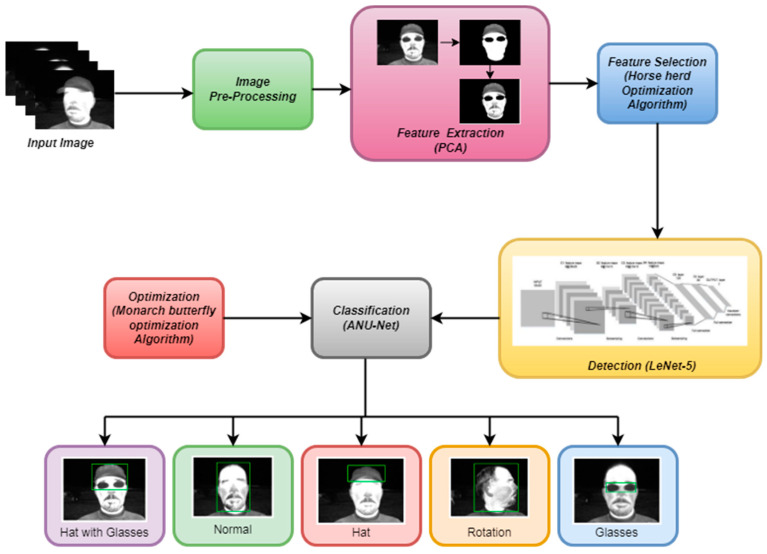
The architecture of the proposed method.

The proposed methodology for classifying a person’s actions and objects using the ANU-Net technique is shown in Figure 2. We proposed a method for detecting and classifying objects and actions such as hats, glasses, normal, rotation, and hat with glasses from human thermal face images, based on deep learning techniques, in this paper. This model is presented in five steps. Initially, the input thermal images should be resized and then converted into grayscale, utilizing the median filter in the pre-processing step. Then, principle component analysis (PCA) is used to extract the features from the pre-processed thermal image in the feature extraction step. Furthermore, as to feature selection, the horse herd optimization algorithm (HOA) is used. After feature selection, the face needs to be detected in order to identify the objects on the face. The LeNet-5 technique is used to detect human faces in thermal images. Finally, classification of the objects and actions on faces is carried out using the ANU-Net approach, combined with the Monarch butterfly optimization algorithm to achieve higher accuracy.

### 3.1. Preprocessing

Initially, the input thermal images should be resized in the pre-processing step. To create a square matrix from the image and make mathematical accounting much simpler, the image was transformed to 256 × 256 pixels by the median filter, then converted to a grayscale image. The purpose of converting true color to grayscale is to improve program computation and simplify the extraction of statistical features for accounting, including mean value and entropy value from the grey-level histogram.

### 3.2. Feature Extraction

After pre-processing, the features are extracted that would be useful in categorizing the images using feature extraction techniques. The extraction of features process is for extracted features which help to detect the face accurately and clearly. In this step, the representative features are extracted with principle component analysis (PCA). The obtaining of features is a significant phase in the model construction process [20]. The principle component technique is used to increase explanation ability while simultaneously reducing the loss of details to reduce the dimension of the dataset being used. The goal of PCA, a mathematical approach that uses a principle component examination, is to reduce the dataset dimension. The process of transforming data into a new coordinate system is known as an orthogonal linear transformation. The native features use linear combinations to offer new features. Applying features with increasing variation is dispatch limiting. Accordingly, the PCA method converts the n vectors {*x*1, *x*2, …, *x*n} from the d-dimensional space to the n vectors {*x*1, *x*2, …, *x*n} in a new d-dimensional space.

(1) $$x^{\prime }i=\sum k=1d^{\prime }ak,iek,d^{\prime }\leq d,$$

Since PCA calculates unit directions, the data can be projected into the input vector. *Y*, where *y* = $d^{t}\bar{\bar{W_{i}}}$ it has a large variance, stands for greater variance. This is written as

(2) $$\sigma_{PCA}(W)=\sigma \left[y^{2}\right]=d^{T}C\bar{\bar{W_{i}}}=\frac{W^{I}CW}{\|{W\|}^{2}}$$

where $\bar{\bar{W}}$ = *W*/‖*W*‖.

Reconstruction of the input data, i.e., $\hat{d}$ use of linear square estimation.

(3) $${\hat{d}}_{t}=\sum_{i=1}^{O}g_{i(t)\bar{\bar{W_{i}}}}$$

With the use of data reconstruction, the error can be recreated and identified by comparing the differences between the original and modified data.

(4) $$e=d-{\hat{d}}_{t}=\sum_{i=O+1}^{n}a_{i}\bar{\bar{W_{\begin{array}{l} i \end{array}}}}$$

To solve this issue requires implementing a novel method for restricting the dimension and enhancing PCA entertainment after overall presentation creation. Based on PCA, the reconstruction error can be reduced [21]. When extending k-dimensional data to subspace, calculations are visible.

PCA reconstruction = PC scores · Eigenvectors T + Mean

According to the proposed method, a maximum likelihood-based model may be used to map the underlying space into the data space.

(5) x=ε+μ+∫Λ

The feasible outcome’s input vector is written as

(6) $$p\left(\varepsilon ;\sigma^{2}\right)={\left(2\pi \sigma^{2}\right)}^{\frac{-p}{2}}\exp\left(\frac{-1}{2}\varepsilon^{\prime }\varepsilon \right)$$

The conditional likelihood will increase by

(7) p(xf;Λ,μ,σ2)=(2πσ2)−2pexp (−12||x−μ−fΛ||2)L(Λ,μ,σ2|x)

(8) $${\left(2\pi \right)}^{{-}_{2}^{np}}|\sum {|}^{{-}_{2}^{n}}\exp\left[-\frac{1}{2}\sum_{i=1}^{n}\left(x_{i}-\mu {)}^{\prime }\sum {}^{-1}(x_{i}-\mu \right)\right]\left(x_{i}-\mu {)}^{\prime }\sum {}^{-1}\right)(x_{i}-\mu )$$

Maximum likelihood observation can also be expressed in $tr\left(\sum {}^{-1}S\right)$ Where *S* can be expressed as,

(9) $$S=\frac{1}{n}\sum_{i=1}^{n}(x_{i}-\hat{\mu })(x_{i}-\hat{\mu }{)}^{\prime }$$

The maximum log-likelihood can be expressed using the equation below.

(10) $$L\left(\Lambda ,\mu ,\sigma^{2}|x\right)=-\frac{np}{2}\log(2\pi )-\frac{n}{2}\log|\sum |-\frac{n}{2}tr\left[\sum {}^{-1}S\right]$$

Maximization of Λ and $\sigma^{2}$ the error can be reduced and yield a better solution based on data reconstruction in PCA. Maximum $\sigma^{2}$ likelihood can be represented below,

(11) $${\hat{\sigma }}^{2}{}_{ML}=\sum_{j=m+1}^{p}\lambda_{j}\times \frac{1}{p-m}$$

The aforementioned model improves output and reduces errors in feature extraction using PCA. These extracted features are used to detect human faces from thermal images.

### 3.3. Feature Selection

Before using detection algorithms, feature selection is an essential step needed to enhance the performance of detection. Multiple features are frequently used to produce good detection results. However, adding more features could result in the so-called “curse of dimensionality”, which impairs detection performance while lengthening computation times and boosting model complexity. As a result, feature selection is required to increase a detection algorithm’s ability to discriminate. By eliminating redundant or unnecessary characteristics, the most important subset of the initial feature set is discovered throughout the feature selection process.

The goal of this work is to use an algorithm to choose features by tackling the feature selection issue. As a result, the horse herd optimization algorithm (HOA) was the main technique employed for this. HOA is a powerful algorithm that takes inspiration from the herding behavior of horses of different ages [22]. HOA performs remarkably well in resolving difficult high-dimensional issues. Its exploration and exploitation efficiency is quite high. It performs better in accuracy and efficiency than several popular metaheuristic optimization techniques. It can identify the optimal solution with the least amount of effort, the least cost, and the least amount of complexity. These behavioral characteristics are frequently seen in horses: hierarchy (H), roam (R), imitation (I), defense mechanism (D), grazing (G), and sociability (S).

The movement of the horses follows Equation (12) at every iteration.

(12) $$X_{m}^{Iter,AGE}={\vec{V}}_{m}^{Iter,AGE}+X_{m}^{(Iter-1),AGE},AGE=\alpha ,\beta ,\gamma ,\delta$$

Every cycle of the algorithm can be described in Equation (13)

(13) $${\vec{V}}_{m}^{Iter,\alpha }={\vec{G}}_{m}^{Iter,\alpha }+{\vec{D}}_{m}^{Iter,\alpha }$$

(14) $${\vec{V}}_{m}^{Iter,\beta }={\vec{G}}_{m}^{Iter,\beta }+{\vec{H}}_{m}^{Iter,\beta }+{\vec{S}}_{m}^{Iter,\beta }+{\vec{D}}_{m}^{Iter,\beta }$$

(15) $${\vec{V}}_{m}^{Iter,\gamma }={\vec{G}}_{m}^{Iter,\gamma }+{\vec{H}}_{m}^{Iter,\gamma }+{\vec{S}}_{m}^{Iter,\gamma }+{\vec{I}}_{m}^{Iter,\gamma }+{\vec{D}}_{m}^{Iter,\gamma }+{\vec{R}}_{m}^{Iter,\gamma }$$

(16) $${\vec{V}}_{m}^{Iter,\delta }={\vec{G}}_{m}^{Iter,\delta }+{\vec{I}}_{m}^{Iter,\delta }+{\vec{R}}_{m}^{Iter,\delta }$$

Grazing: Horses are grazing creatures that eat vegetation such as grass and other forages. Their daily grazing time ranges from 16 to 20 h, and they take only brief breaks. Equations (17) and (18) correspond to how grazing is represented mathematically:

(17) $${\vec{G}}_{m}^{Iter,AGE}=g_{Iter}(\overset{⌣}{u}+\rho \overset{⌣}{l})[X_{m}^{\left(Iter-1\right)}]$$

(18) $$g_{m}^{Iter,AGE}=g_{m}^{(Iter-1),AGE}\times \omega_{g}$$

With each repetition, this factor brings linearity down by *ω_g_*. P is a chance number between 0 and 1, $\overset{⌣}{u},$ and $\overset{⌣}{l}$ are the respective lower and upper limits of the grazing space.

Hierarchy (H): Horses cannot exist in complete freedom. They go through life following a leader, which is frequently observed in humans. By the law of hierarchy, an adult stallion is also in charge of providing authority within wild horse herds. Equation (19) can be used to describe this.

(19) $${\vec{H}}_{m}^{Iter,AGE}=h_{m}^{Iter,AGE}[X_{•}^{\left(Iter-1\right)}-X_{m}^{\left(Iter-1\right)}]$$

(20) $$h_{m}^{Iter,AGE}=h_{m}^{\left(Iter-1\right)}\times \omega_{h}$$

where ${\vec{H}}_{m}^{Iter,AGE}$ shows where the best horse is located $X_{•}^{\left(Iter-1\right)}$ and how the best horse’s placement affects the velocity parameter.

Sociability (S): Horses need to interact with people and occasionally coexist with other animals. Pluralism makes it easier to leave and enhances the likelihood of survival. Due to their social characteristics, you may frequently observe horses fighting with one another, and a horse’s singularity is what makes them so irritable. The herd described in the following equation attracts the attention of horses between the ages of 5 and 15 years, as is evident from observation:

(21) $${\vec{S}}_{m}^{Iter,AGE}=S_{m}^{Iter,AGE}\left[(\frac{1}{N}\sum_{j=1}^{N}X_{j}^{\left(Iter-1\right)})-X_{m}^{\left(Iter-1\right)}\right]\;\mathrm{AGE}=\beta ,\gamma$$

(22) $${\vec{S}}_{m}^{Iter,AGE}=S_{m}^{Iter}\left[\left(\frac{1}{N}\sum_{j=1}^{N}X_{j}^{\left(Iter-1\right)}\right)-X_{m}^{\left(Iter-1\right)}\right]\;\mathrm{AGE}=\beta ,\gamma$$

(23) $$S_{m}^{Iter,AGE}=S_{m}^{(Iter-1),AGE}\times \omega_{s}$$

N stands for the total number of horses, and AGE stands for the age range of each horse. In the evaluation of the sensitivity parameter, the s coefficient for horse’s *β* and *γ* is determined.

Imitation: Horses mimic one another and learn from one another’s desirable and unattractive behavior, including where to find the best pasture. The imitation behavior of horses is likewise considered a factor in the current approach.

(24) $${\vec{I}}_{m}^{Iter,AGE}=i_{m}^{Iter,AGE}\left[\left(\frac{1}{pN}\sum_{j=1}^{pN}{\hat{X}}_{j}^{\left(Iter-1\right)}\right)-X^{\left(Iter-1\right)}\right],\;\mathrm{AGE}=\gamma$$

(25) $$i_{m}^{Iter,AGE}=i_{m}^{Iter,AGE}\times \omega_{i}$$

As the number of horses in the best places, *pN* is indicated. Ten percent of the horses is the recommended value for *p*.

Defense mechanism (D): Horses’ behavior is a result of their experiences as prey animals. They exhibit the fight-or-flight reaction to defend themselves. Their initial response is to run away. Additionally, they buck upon being trapped. For sustenance, so they can remove rivals, and to avoid dangerous areas where wolves and other natural predators are present, horses engage in battle.

Equations (26) and (27) show the horse’s defense mechanism, which is a negative coefficient, to prevent the animal from being in the wrong positions.

(26) $${\vec{D}}_{m}^{Iter,AGE}=-d_{m}^{Iter,AGE}\left[\left(\frac{1}{qN}\sum_{j=1}^{qN}{\overset{⌣}{X}}_{j}^{\left(Iter-1\right)}\right)-X^{\left(Iter-1\right)}\right]$$

(27) $$d_{m}^{Iter,AGE}=d_{m}^{(Iter-1),AGE}\times \omega_{d}$$

The number of horses in the most underdeveloped areas is also shown by *qN*. A 20% horse population is thought to be equivalent to *q*.

Roam (R): In the retrieval of food, wild horses graze and travel around the countryside from pasture to pasture. Although they still have this quality, most domestic horses are kept in stables. A factor r displays this tendency and simulates it as a random movement. Young horses nearly always exhibit roaming, which eventually decreases as they mature.

(28) $${\vec{R}}_{m}^{Iter,AGE}=r_{m}^{Iter,AGE}\rho X^{\left(Iter-1\right)},\;\mathrm{AGE}=\gamma ,\delta$$

(29) $$r_{m}^{Iter,AGE}=r_{m}^{(Iter-1),AGE}\times \omega_{r}$$

Horse speed *δ* when it is between 0 and 5 years old:

(30) $$\begin{array}{ll} {\vec{V}}_{m}^{Iter,\delta }= & \left[g_{m}^{(Iter-1),\delta }\omega_{g}\left(\overset{⌣}{u}+\rho \overset{⌣}{l}\right)\left[X_{m}^{\left(Iter-1\right)}\right]\right]+\left[i_{m}^{Iter-1,\delta }\omega_{i}\left[\left(\frac{1}{pN}\sum_{j=1}^{pN}{\hat{X}}_{j}^{Iter-1}\right)-X^{Iter-1}\right]\right]+\\ & \left[r_{m}^{Iter-1,\delta }\omega_{r}pX^{Iter-1}\right] \end{array}$$

Horses’ speeds *γ* between the ages of 5 and 10:

(31) $$\begin{array}{ll} {\vec{V}}_{m}^{iter.\gamma }= & \left[g_{m}^{Iter-1.\gamma }\omega_{g}\left(\overset{⌣}{u}+\rho \overset{⌣}{l}\right)\left[X_{m}^{Iter-1}\right]\right]+\left[h_{m}^{Iter-1.\gamma }\omega_{h}\left[X_{*}^{Iter-1}-X_{m}^{Iter-1}\right]\right]+\\ & \left[s_{m}^{Iter-1.\gamma }\omega_{s}\left[\left(\frac{1}{N}\sum_{j=1}^{N}X_{j}^{Iter-1}\right)-X_{m}^{Iter-1}\right]\right]+\left[i_{m}^{Iter-1.\gamma }\omega_{i}\left[\left(\frac{1}{pN}\sum_{j=1}^{pN}{\overset{⌢}{X}}_{j}^{Iter-1}\right)-X^{Iter-1}\right]\right] \end{array}$$

Horses’ speeds *β* between 10 and 15 years old:

(32) $$\begin{array}{ll} {\vec{V}}_{m}^{Iter.\beta }= & \left[g_{m}^{Iter-1.\beta }\omega_{g}\left(\overset{⌣}{u}+\rho \overset{⌣}{l}\right)\left[X_{m}^{Iter-1}\right]\right]+\left[h_{m}^{Iter-1.\beta }\omega_{h}\left[X_{*}^{Iter-1}-X_{m}^{Iter-1}\right]\right]\\ & +\left[s_{m}^{Iter-1.\beta }\omega_{s}\left[\left(\frac{1}{N}\sum_{j=1}^{N}X_{j}^{Iter-1}\right)-X_{m}^{Iter-1}\right]\right]-\left[d_{m}^{Iter-1.\beta }\omega_{d}\left[\left(\frac{1}{qN}\sum_{j=1}^{qN}{\overset{⌣}{X}}_{j}^{Iter-1}\right)-X^{Iter-1}\right]\right] \end{array}$$

Older-than-15-years-old horses’ top speeds *α*:

(33) $$\begin{array}{ll} {\vec{V}}_{m}^{Iter.\alpha }= & \left[g_{m}^{Iter-1,\alpha }\omega_{g}\left(\overset{⌣}{u}+\rho \overset{⌣}{l}\right)\left[X_{m}^{Iter-1}\right]\right]-\\ & \left[d_{m}^{Iter-1,\alpha }\omega_{d}\left[\left(\frac{1}{qN}\sum_{j=1}^{qN}{\overset{⌣}{X}}_{j}^{Iter-1}\right)-X^{Iter-1}\right]\right] \end{array}$$

The outcomes validated HOA’s capacity to deal with complex situations, such as several uncertain variables in high-dimensional areas. Adult *α* horses begin a highly precise local search in the vicinity of the global optimum. They exhibit a tremendous desire to explore new terrain and locate new global spots. Young *δ* horses provide ideal candidates for the random search phase due to certain behavioral traits they possess.

### 3.4. Detection

After the feature selections, the face detection process is to separate the image into background and foreground. In the detection step, face identification is more important to the classification process, as here we classify the objects and actions on the face. The detection process used LeNet-5 to execute detection in this research. The LeNet-5 technique is utilized with images where the background and foreground colors of images are very close. The LeNet-5 technique is described below.

Effective deep skin lesions are detected by using this detection network. According to the needs of the application, many kinds of CNNs can be employed for detection, and faces can be detected by using a LeNet-5 pre-trained network on the ImageNet database. LeNet-5 has seven layers: an input layer, two pooling layers, a fully connected layer, two convolutional layers, and an output layer [23]. LeNet-5’s detailed architecture is shown in Table 1. Several weighted layers in LeNet-5 are built on the concept of eliminating the convolution layer blocks by leveraging shortcut connections. The fundamental building blocks are referred to as “bottleneck” blocks, two design rules are used by these blocks: The same output feature size is produced by using the same number of filters. The convolution layers down-sample at a rate of two strides per layer. Before the activation of rectified-linear-unit (ReLU) and after each convolution, batch normalization is conducted.

The final detection network uses these region proposals for object classification. Anchor boxes are originally produced across each feature map pixel with varying scales and aspect ratios in the RPN. Nine anchor boxes are typically utilized, with aspect ratios of 1:1, 1:2, and 2:1 and scales of 128, 256, and 512. An anchor box’s probability of containing a background or object is predicted by RPN. The required object proposals are sent to the next stage in the form of a list of filtered anchor boxes. Equations (34) and (35) must be used to convert the final predicted region proposals from the anchor boxes. The translation between the center coordinates that is scale-invariant is shown in Equation (34). Equation (35) shows how the height and width translate in log space.

(34) $$V_{x}=\frac{x_{p}-x_{a}}{w_{a}},\;V_{y}=\frac{y_{p}-y_{a}}{h_{a}}$$

(35) $$V_{w}=\log\left(\frac{w_{p}}{w_{a}}\right),\;V_{h}=\log\left(\frac{h_{p}}{h_{a}}\right)$$

where the bounding box regression vectors are represented by $V_{x}$$V_{y}$$V_{w}$, and $V_{h}$, and coordinates for the height, width, and center in x and y are depicted by *h*, *w*, and *x*, *y*. Additionally, $x_{a}$$x_{p}$ are the corresponding centers of the anchor box and proposal box. The convolutional layers and fully connected layers are utilized in this process to detect the human face in thermal images based on extracted features.

### 3.5. Classification

We classify the objects and actions from the detected face in thermal images. So, for the classification of thermal images, we create an integrated network termed Attention U-Net++. This is a better classification technique compared with other existing classification techniques and is also novel and reasonable. A sequence of U-Nets with various depths is integrated using nested U-Net architecture, which takes from DenseNet. The nested framework is distinctive from U-Net, and used nested convolutional blocks and redesigned dense skip links among the encoder and decoder at various depths [24]. In layered U-Nets, each nested convolutional block captures semantic information using many convolution layers. Additionally, each layer in the block is linked together via connections, allowing the concatenation layer to combine semantic data of various levels.

#### 3.5.1. Attention Gate (AG)

The AG employs the PASSR net’s model and includes an effective attention gate into nested architecture. Here is a more thorough analysis of the attention gate:

To facilitate learning of the subsequent input, the initial input (*g*) serves as the gating signal.

- As a gating signal to facilitate the learning of the next input, the first input (*g*) is used (*f*). In other words, this gating signal (*g*) can choose more advantageous features from encoded features (*f*) and transfer them to the top decoder.
- These input data are combined pixel by pixel following a CNN operation (*W_g_*, *W_f_*) and batch norm (*b_g_*, *b_f_*).
- The S-shaped activation function sigmoid $\left(\sigma^{2}(x)=\frac{1}{1+e^{\left(-x\right)}}\right)$ is chosen to obtain the attention coefficient (*α*) and to perform the divergence of the gate’s parameters.
- The result can be produced by multiplying each pixel’s encoder feature by a certain coefficient.

Following is a formulation of the attention gate feature selection phase:

(36) $$F=\sigma_{1}[(W_{f}^{T}\times f+b_{f})+(W_{g}^{T}\times g+b_{g})]$$

(37) $$\alpha =\sigma_{2}(W_{\theta }^{T}\times F+b_{\theta })$$

(38) output=f×α

The AG can learn to classify the task-related target region, and it can suppress the task-unrelated target region. This work incorporates the attention gate to enhance the effectiveness of propagating semantic information through skip links in the innovative proposed network.

#### 3.5.2. Attention-Based Nested U-Net

Based on the attention mechanism and the nested U-Net architecture, ANU-Net is an integrated network for thermal image classification. In ANU-Net, which employs nested U-Net as its primary network design, hierarchical traits can be recovered that are more useful. Through the extensive skip connections, the encoder transmits the context information collected to the relevant layers decoder. By using the proposed ANU-Net classification model, the time complexity is reduced, effective training is performed, and the performance of the classification is improved.

When there are several dense skip connections, each convolutional block in the decoder obtains two equal-scale feature maps as inputs: The outcomes of earlier attention gates with residual connections at the same depth are used to create the preliminary feature maps, and the output of the deeper block deconvolution process is used to create the final feature map. After receiving and concatenating all extracted feature maps, the decoder reconstructs features from the bottom up.

The extracted feature map from ANU-Net may be expressed as follows: Let $X_{i,j}$ indicate the outcome of the convolutional block, while i defines the feature depth and j signifies the sequence of the convolution block.

(39) $$X_{i,j}=\{\begin{array}{ll} \Phi [X_{i-1,j}], & j=0\\ \Phi [\sum_{k=0}^{j=1}Ag(X_{i,k}),Up(X_{i+1,j-1})],\; & j>0 \end{array}$$

$Up(X_{i+1,j-1})$ and $Ag(X_{i,k})$ the attention gate and mean up-sampling selection accordingly, $\sum_{k=0}^{j=1}Ag(X_{i,k})$ indicate that concatenate the outcome of the attention gates from node *X_i_*, *k* = 0 to *X_i_*, *k* = *j* − 1 in the *i*th layer.

Only the encoder’s selected same-scale feature maps will be used by the decoder’s convolution blocks after the concatenation procedure, rather than all of the feature maps that were obtained via dense skip connections. The outcome of the j preceding blocks in this layer serves as inputs, while block *X*_1_’s up-sampling feature in the second layer serves as additional input. Two of ANU-Net key breakthroughs are the network transfer of features collected from the encoder. Additionally, the attention gate is implemented in the decoder path in between layered blocks retrieved at various layers and can be combined with a targeted selection. ANU-Net accuracy should therefore be increased as a result. The accuracy of the classification technique has not made more changes to other existing techniques. So, the monarch butterfly optimization (MBO) algorithm is utilized with ANU-Net to improve classification accuracy.

### 3.6. Monarch Butterfly Optimization (MBO) Algorithm

We included the optimization process to obtain better classification accuracy in this paper. Using the optimization algorithm can achieve higher accuracy and better outcomes in the classification step. Therefore, we utilized the Monarch butterfly optimization (MBO) algorithm, accompanied by a classification technique. The MBO is an algorithm based on a population that is thought to fall under swarm intelligence algorithms. These algorithms are motivated by the behavior of a few insects that tend to swarm, such as butterflies, bees, etc. As previously said, the MBO was created recently. The creators of this algorithm received their inspiration from a species of butterfly which is unique to North America and distinguished by its lovely form, which features black and orange colors [25]. Various optimization issues are resolved by simulating the migratory behavior of these butterflies. To obtain the best answer to the issue, several guidelines and fundamental ideas must be followed:

1. Either Land 1 or Land 2 is home to all of the butterflies (the home after migration).
2. No matter if the parents are in Land 1 or Land 2, the migration operator generates each butterfly’s offspring.
3. Out of the parent and offspring, one of the two will be eliminated by a candidate function because the population should not change and should be constant.
4. The butterflies chosen using the candidate function are handed down to the following generation without being altered by the migration operator.

#### 3.6.1. Migration Facilitator

The relocation procedure of the butterflies is represented as follows:

(40) $$X_{i.k}^{t+1}=X_{r1.k}^{t}$$

where $X_{r1.k}^{t}$ denotes the Kth components of the newly generated location and $X_{i.k}^{t+1}$ denotes the Kth components of *Xi* at the *t* + 1 generation, which convey the place of a butterfly *i*. In this case, the random number *r* was generated using the following equation:

(41) R=rand×peri

where *peri* stands for the time during the migration.

The following equation is used to determine the Kth components of the next-generation location:

(42) $$X_{i.k}^{t+1}=X_{r2.k}^{t}$$

where $X_{r2.k}^{t}$ denotes the butterfly *r*2’s Kth generation of *X_r_*_2_’s elements. Consequently, *P* stands for the number of monarch butterflies per acre.

#### 3.6.2. Butterfly Adjusting Operator

The following butterfly adjustment operator can also be utilized to obtain the monarch butterflies’ locations in addition to the migration operator. The following sentence adds the butterfly adjusting operator method. If an oddly produced number rand is less than or equal to p for all the components in monarch butterfly *j*, it can be improved as

(43) $$X_{j.k}^{t+1}=X_{best.k}^{t}$$

This $X_{best.k}^{t}$ denotes the kth component of $x_{best}$ that is the best monarch butterfly in Lands 1 and 2. t is the generation number currently in effect. The update can be made, however, if rand is greater than p.

(44) $$X_{j.k}^{t+1}=X_{r3.k}^{t}$$

where $X_{r3.k}^{t}$ denotes the randomly chosen kth element from *X_r_*_3_ in Land 2. In this case, $r3\in \left\{1,2,\ldots ,NP_{2}\right\}$. If rand > BAR in this scenario, it can then be updated further as shown below.

(45) $$X_{j.k}^{t+1}=X_{j.k}^{t+1}+\alpha \times \left(dx_{k}-0.5\right)$$

where BAR is the rate at which a butterfly adjusts. Levy flight can be used to determine the monarch butterfly’s walk step or *dx*.

(46) $$dx=Levy\;\left(x_{j}^{t}\right)$$

In Equation (45), the weighting factor is represented by α which is obtained in Equation (47).

(47) $$\alpha =S_{max}/t^{2}$$

where *t* is the current generation and $S_{max}$ is the maximum distance a monarch butterfly individual may travel in one step. The larger, denoting a lengthy step in the search process, increases the influence of dx on $x_{j.k}^{t+1}$ and promotes the exploration process, whereas the little, denoting a short step in the search process, lessens the influence of *dx* on $x_{j.k}^{t+1}$ and promotes the exploitation phase. As a result, it can describe the person who adjusts the butterfly. Using MBO with the classification technique gives a higher classification accuracy to our experimental outcomes.

## 4. Results and Discussion

This section begins by comparing our method to other existing approaches for recognizing faces and categories of objects and actions in thermal images from the Terravic Facial Infrared Database. The subsections that follow give the assessment results based on experimental data to evaluate our methodology.

### 4.1. Dataset Description

The Terravic Facial IR Database was utilized to assess this strategy. The dataset collection consists of 20 classes, each of which is represented by a series of greyscale images (360 × 240). As three classes were corrupted, we used 17 classes in this paper. A total of 200 greyscale images were utilized for each class. The images are 8-bit grayscale JPEG files and 320 × 240 pixels in size. The distribution of the images for each class is shown in Table 2.

### 4.2. Evaluation Metrics

Each detector generates a bounding box that shows the location of people’s faces in the input thermal images. We can identify which predicted bounding boxes are accurate by comparing them to ground truth boxes. The intersection over union is used to quantify overlap, which calculates the ratio between a pair of boxes’ union and intersection. A perfect disjoint alignment is 0, and a perfect alignment of the two boxes is 1. A bounding box is considered to be an accurate forecast if it overlaps the ground truth by at least 0.3. Typically, an overlap of 0.5 or greater is necessary for object detection. However, it was chosen to be a little leaner here because some of these things can be rather little. Only one prediction can be mapped to a ground truth box. A prediction is deemed accurate if it falls within a ground truth box; otherwise, it is deemed incorrect.

In terms of performance measures, we looked at the proposed method’s precision (P), accuracy (A), F1-score (F), and recall (R). These metrics indicate:

#### 4.2.1. Accuracy

The accuracy measure is computed to identify the correctness of advertisement classification.

(48) $$Accuracy=\frac{TP+TN}{TP+TN+FP+FN}$$

#### 4.2.2. Precision

Precision is defined as the ratio of precisely anticipated positive occurrences to all anticipated positive observations. *Precision* is the capacity to do the following things:

(49) $$Precision=\frac{TP}{TP+FP}$$

#### 4.2.3. Recall

The true positive rate (TPR) and sensitivity are both terms for recall. The recall score reflects the classifier’s ability to locate all positive samples. It is the total of *TP* and *FN* divided by *TP*. It can be described in the following terms:

(50) $$Recall=\frac{TP}{TP+FN}$$

#### 4.2.4. F-Measure

F-Measure determines the harmonic mean of recall and precision.

(51) $$F1\;Score=2\times \frac{precision\times recall}{precision+recall}$$

### 4.3. Quantitative Evaluation

**Figure 3:**
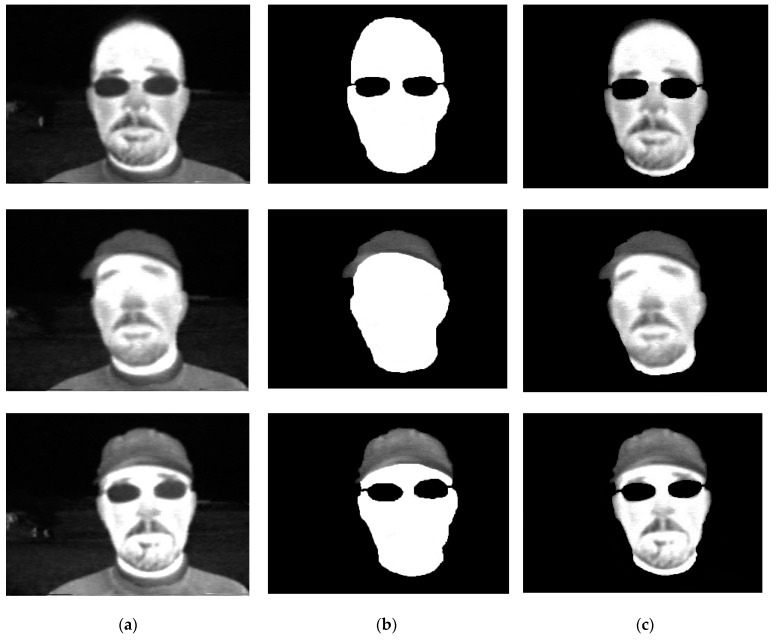
Extracting the faces. (**a**) Original Image. (**b**) Feature Extraction. (**c**) Face Extraction.

The experimental results show the extraction of faces and classification outcomes. We proposed a method for the detection and classification of human thermal face objects and actions based on different attributes (hat, glasses, normal, rotation, and hat with glasses) using various deep-learning models. Initially, we resized the input images and then gray scaled the images by using the median filter in the pre-processing step. In addition, we extracted the features from pre-processed thermal images using the principle component analysis (PCA) technique to detect the face accurately. Furthermore, the horse herd optimization algorithm (HOA) was utilized to select the features. Then, to detect human faces in the thermal images, the LeNet-5 technique was employed. Face detection was needed to classify the images beforehand. Finally, we classified the objects on faces by using the ANU-Net technique. To obtain a higher classification accuracy, we used the MBO optimization technique. Here, we provide some thermal images which were collected from our database to detect the faces in a single thermal image and also classify the faces by emotions. Figure 3 shows the results of face extraction outcomes using the PCA technique.

Using the principle component analysis technique, we extracted the features of different variations from the thermal input images and then extracted the faces, utilizing the features. These are all extracted things that make the classification of objects and actions and detection of face objects accurate.

**Figure 4:**
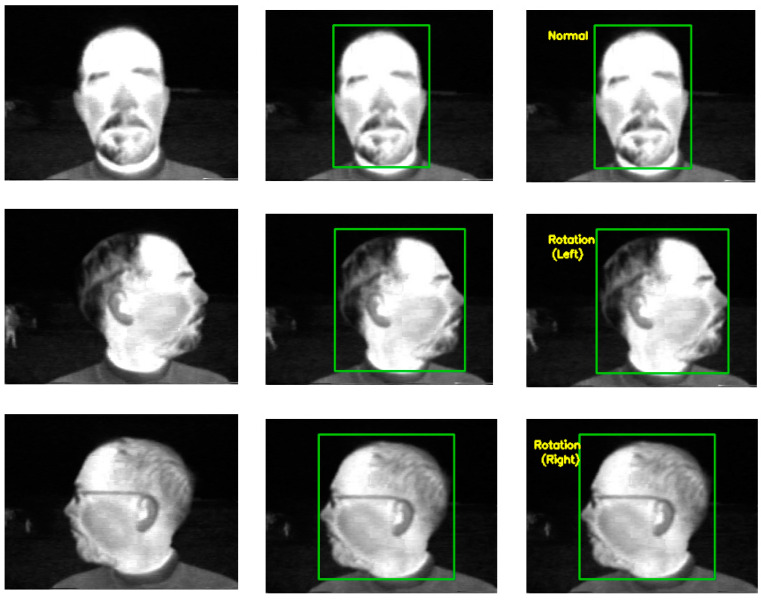

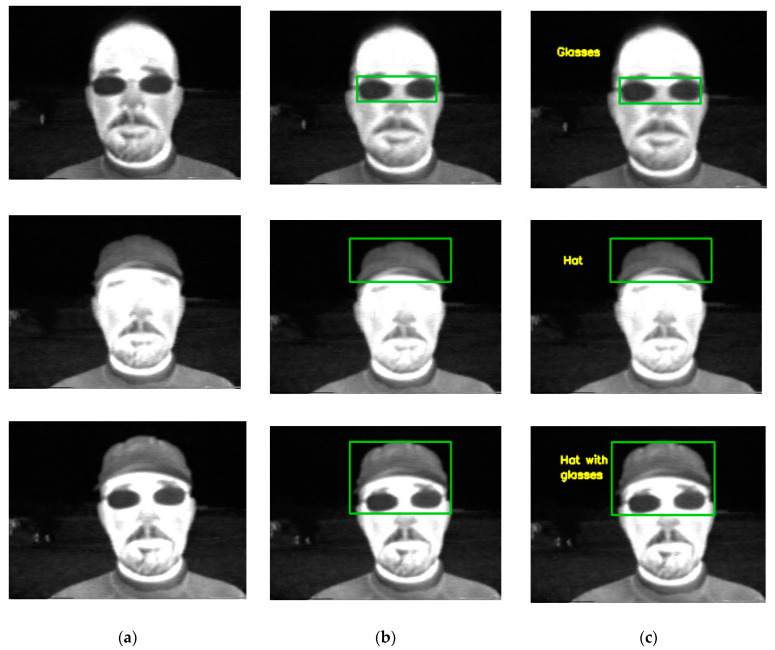
Classification of objects and actions. (**a**) Original Image. (**b**) Objects and Action Detection. (**c**) Classification results.

The detection of face objects and actions is shown in Figure 4. The LeNet-5 technique provides accurate recognition of things on thermal human face images. Additionally, Figure 4 displays the outcome of classification as a boundary box with the category names.

### 4.4. Performance Metrics

**Figure 5:**
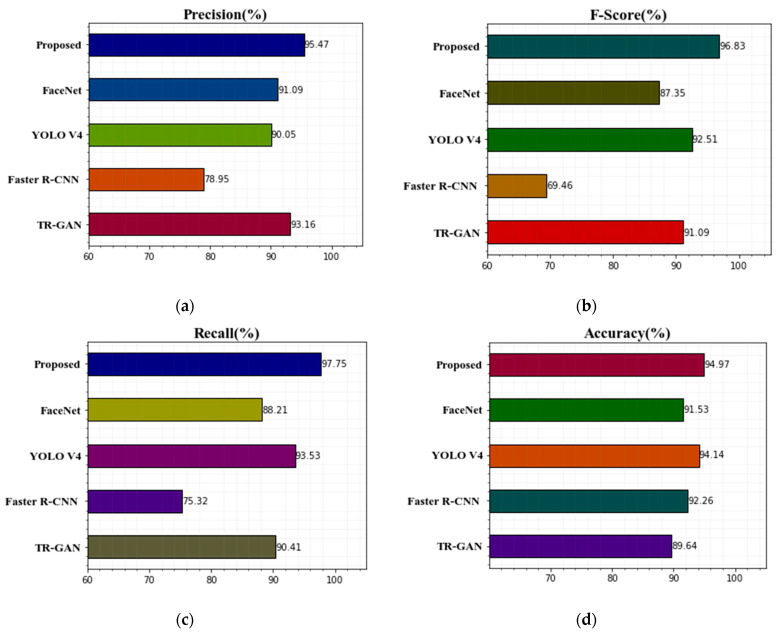
Comparison of the suggested technique’s classification results with previous methods without optimization algorithm (**a**) precision, (**b**) F-score, (**c**) recall, and (**d**) accuracy.

We compared various approaches with the Terravic Facial Infrared Database studies. Our technique had the highest effectiveness and increased the recognition rate. We had no problems identifying a single face in thermal images opposing a dark foreground. Here, our technique obtained the highest accuracy compared with other techniques. This paper used an optimization algorithm for better classification results with classification techniques. We presented the without-optimization algorithm and with-optimization comparison results as a graph. Our approach was still able to identify the thermal face regardless of the head’s rotation, glasses, hat, normal, and hat with glasses. The proposed approach operates nicely in our database, according to the results. Table 3 shows our findings based on accuracy, precision, recall, and F-score without an optimization algorithm. Figure 5 shows the comparison of the proposed technique’s classification results with other existing techniques without optimization algorithm graphs for precision, F-score, recall, and accuracy. It gives lower classification accuracy values. Therefore, we used an optimization technique to improve the accuracy level in our experiments.

**Figure 6:**
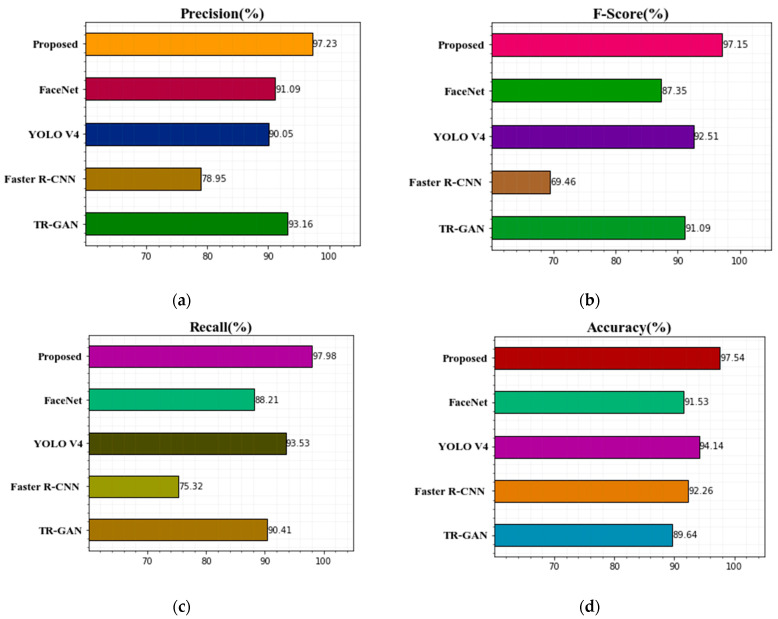
Comparison of the suggested technique’s classification results with previous methods with optimization algorithm (**a**) precision, (**b**) F-score, (**c**) recall, and (**d**) accuracy.

Table 4 shows our findings based on accuracy, precision, recall, and F-score without an optimization algorithm. Figure 6 shows the comparison of the proposed technique’s classification results with other existing techniques with optimization algorithm graphs for precision, F-score, recall, and accuracy. Table 4 shows the results for TR-GAN [19], Faster R-CNN [17], YOLO V4 [26], FaceNet [27], and the proposed ANU-Net with MBO technique on the Terravic Facial IR Database in terms of recall, precision, F-score, and precision. Based on the results, we can see that the proposed methodology has higher classification accuracy values than other existing deep learning approaches in terms of recognition rate accuracy, recall, precision, and F-score. The achieved higher F-score for the proposed technique is 97.15%, compared to 91.09% for TR-GAN [19], 69.46% for Faster R-CNN [17], 92.51% for YOLO V4 [26], and 87.35% for FaceNet [27]. The proposed method’s acquired precision is quite similar to SSD’s precision. Additionally, when compared to other existing methodologies, the proposed approach’s recall is superior. TR-GAN [19] has the lowest accuracy rate, at 89.64 percent. The comparison of the proposed method classification results with other methods with optimization algorithm graphs for precision, F-score, recall, and accuracy is displayed in Figure 6. Compared with other existing methods, the proposed ANU-Net technique achieves higher accuracy with the optimization algorithm. From the experiment analysis, the performance of the classification improved by using the proposed model, and computation time is reduced for training the images.

Figure 6 shows how the ANU-Net improves the classification outcomes. It obtains an F-score of 97.15 percent, recall of 97.98 percent, precision of 97.23 percent, and accuracy of 97.54 percent.

### 4.5. Evaluation of Training Results

**Figure 7:**
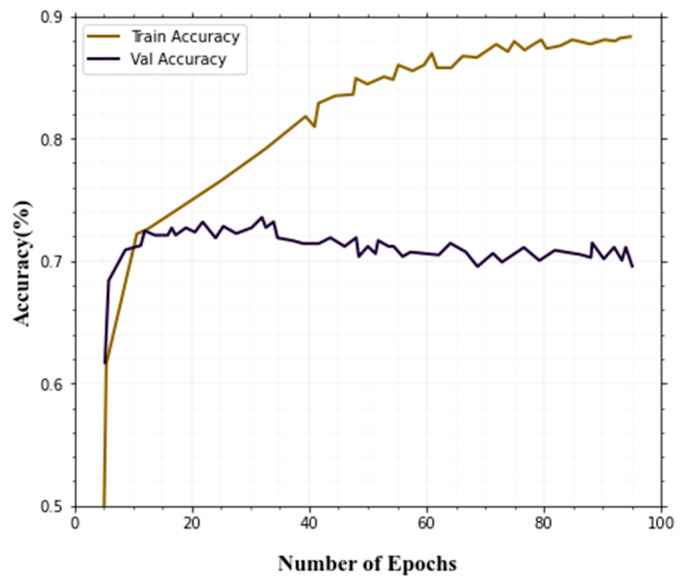
Training and validation accuracy.

Training accuracy and validation accuracy curves converge in the end, and after 100 epochs achieved an accuracy of 97.54%, which is quite good. Figure 7 shows the training and validation accuracy.

**Figure 8:**
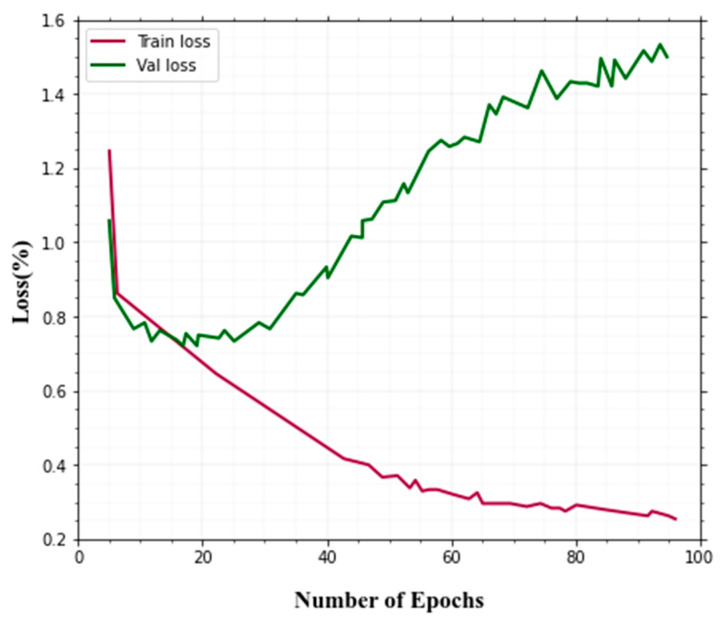
Training and validation loss.

The validation loss curve jumps up and down a bit. However, because the curve does not rise over epochs and the variance between testing and training loss is small, this could be acceptable. Figure 8 shows the training and validation loss.

The accuracy and loss during training are shown in Figure 7 and Figure 8. The ANU-Net is providing better accuracy and loss predictions. Compared to other techniques, our method is given better performance in the training and validation process for the classification of thermal image objects and actions.

### 4.6. Computation Time

**Figure 9:**
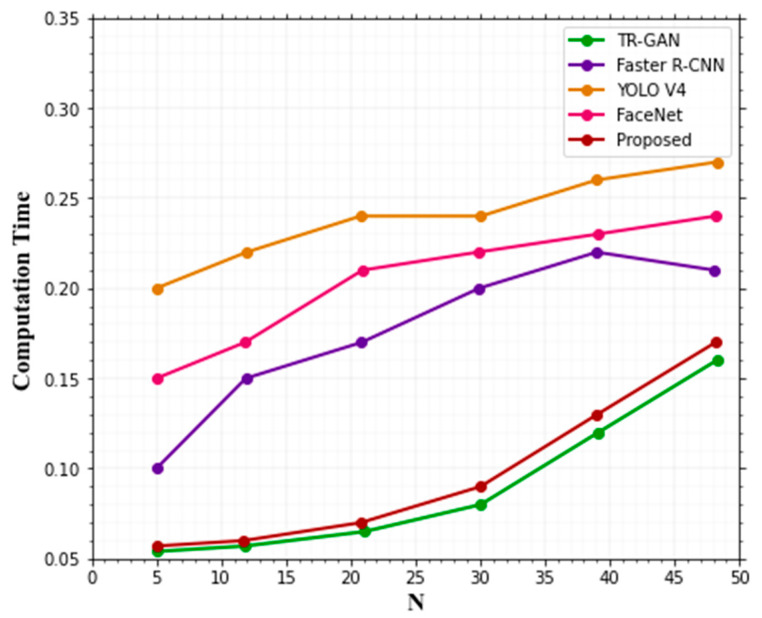
Comparing the time complexity of the suggested approach to the existing techniques.

Computation time is another factor that is compared. Deep learning methods aim to solve the complexity of computation. Using our proposed ANU-Net technique gives less computational time compared with other existing techniques’ computation time, shown in Table 5. It gives better classification accuracy with less computational time. Figure 9 displays how long a computational time is required for the state-of-the-art methods and the proposed model using the Terravic Facial IR Database.

From Figure 9, it can be shown that the proposed strategy exceeded the other techniques and displayed less computation time.

**Figure 10:**
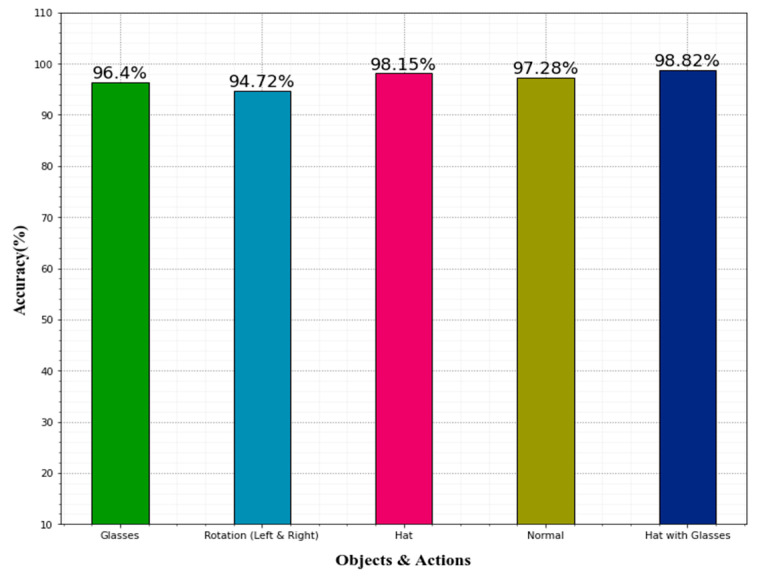
Evaluation of the classifier’s performance in analyzing emotions in terms of accuracy.

A comparison of accuracy in detecting various human facial emotions is depicted in Table 6. The various face objects and actions include glasses, rotation (left and right), hat, normal, and hat with glasses. The glasses yielded 96.4% accuracy, rotation (left and right) obtained 94.72% accuracy, the hat obtained 98.15% accuracy, normal yield 97.28% accuracy, and the hat with glasses achieved 98.82% accuracy. When observing accuracy among the five objects on the human face, the hat with glasses achieved highest accuracy. Figure 10 shows the graphical representation of the objects and actions on the human face classifications.

A confusion matrix is used to describe how well a classification method performs. The output of a classification method is shown and presented in a confusion matrix.

**Figure 11:**
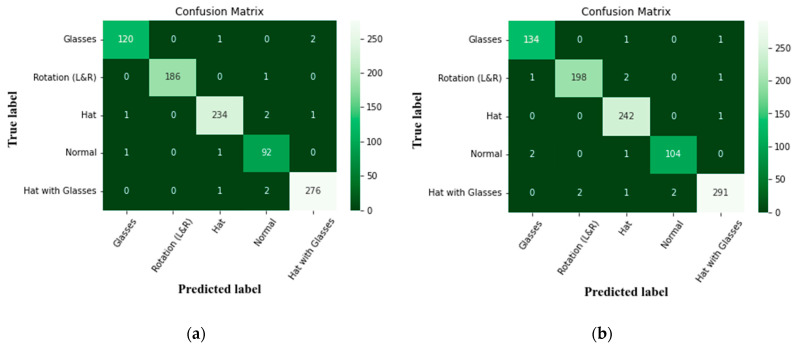
Confusion matrix of face objects (**a**) without optimization, and (**b**) with optimization.

The objects and actions including hats, glasses, normal, rotation (left and right), and hats with glasses are the ones that achieved less accurate precision scores in the model, which are shown in Figure 11.

## 5. Conclusions

Face recognition has gained popularity over the past few years. However, face detection in thermal images is challenging in a variety of positions and lighting conditions. We proposed a method for detecting and classifying human thermal facial objects based on multiple deep learning models, including hat, glasses, normal, rotation, and hat with glasses in this paper. Initially, we resized the images and converted an input image into a grayscale image using the median filter. In addition, we extracted the features from thermal images utilizing principle component analysis (PCA). Furthermore, the horse herd optimization algorithm (HOA) was utilized to select the features. Then, to detect human faces in the thermal image process, the LeNet-5 technique was employed. Finally, we classified the objects on faces by using the ANU-Net technique with the Monarch butterfly optimization (MBO) algorithm to improve accuracy. Our model performs optimally for objects and actions classification from thermal images, according to the Terravic Facial IR Database results. These experimental results, achieving high performance in the evaluation, show 97.54% of classification accuracy with an optimization technique. The test on the database evaluates the usefulness of the proposed method. In the future, we will gradually improve the model and further improve the classification accuracy of this algorithm, based on considerations of how to improve its high-level features.

## Figures and Tables

**Table 1:** LeNet-5 detection network.

Layers	Name of the Layers	Input Size	Output Size	Pooled Area	Convolution Kernel Size	Step Size
Input	Input layer	32 × 32	28 × 28		5 × 5	1
Layer1	Convolutional layers	6@28 × 28	6@14 × 14	2 × 2		2
Layer 2	Pooled layers	6@14 × 14	16@10 × 10		5 × 5	1
Layer 3	Convolutional layers	16@10 × 10	16@5 × 5	2 × 2		2
Layer 4	Pooled layers	16@5 × 5	120@1 × 1		5 × 5	1
Layer 5	Fully connected layer	1 × 120	1 × 84			
Layer 6	Fully connected layer	1 × 84	1 × 7			
Output	Output layer	1 × 7				

**Table 2:** Database image distribution.

**Face Class**	Face 01	Face 02	Face 03	Face 04	Face 07	Face 08	Face 09	Face 10	Face 11
**Images/Class**	227	620	592	487	1297	857	1117	283	434
**Face Class**	Face 12	Face 13	Face 14	Face 15	Face 16	Face 17	Face 18	Face 19	Face 20
**Images/Class**	2179	1417	1482	1125	1611	2632	2215	2539	1670

**Table 3:** Using the proposed and compared approaches, calculate precision, F-score, accuracy, and recall (%) without optimization.

Approaches	Precision	F-Score	Accuracy	Recall
TR-GAN [19]	93.16	91.09	89.64	90.41
Faster R-CNN [17]	78.95	69.46	92.26	75.32
YOLO V4 [26]	90.05	92.51	94.14	93.53
FaceNet [27]	91.09	87.35	91.53	88.21
Proposed (ANU-Net)	95.47	96.83	94.97	97.75

**Table 4:** Using the proposed and compared approaches, calculate precision, F-score, accuracy, and recall (%) while with optimization.

Approaches	Precision	F-Score	Accuracy	Recall
TR-GAN [19]	93.16	91.09	89.64	90.41
Faster R-CNN [17]	78.95	69.46	92.26	75.32
YOLO V4 [26]	90.05	92.51	94.14	93.53
FaceNet [27]	91.09	87.35	91.53	88.21
Proposed + Optimized (ANU-Net)	97.23	97.15	97.54	97.98

**Table 5:** Using the proposed and compared approaches, with optimization.

Approaches	Computation Time (ms)
TR-GAN [19]	0.16
Faster R-CNN [17]	0.21
YOLO V4 [26]	0.27
FaceNet [27]	0.24
Proposed (ANU-Net)	0.17

**Table 6:** Comparisons of various human face emotions accuracy.

Face Emotions	Accuracy (%)
Glasses	96.4
Rotation (Left & Right)	94.72
Hat	98.15
Normal	97.28
Hat with Glasses	98.82

## Data Availability

Not applicable.
